# Retrospective Analysis of Risk Factors for Recurrence of Chronic Subdural Haematoma after Surgery

**DOI:** 10.3390/jcm13030805

**Published:** 2024-01-30

**Authors:** Samer Zawy Alsofy, Marc Lewitz, Kyra Meyer, Thomas Fortmann, Eike Wilbers, Makoto Nakamura, Christian Ewelt

**Affiliations:** 1 Department of Medicine, Faculty of Health, Witten/Herdecke University, 58448 Witten, Germany; mlewitz@barbaraklinik.de (M.L.); tfortmann@barbaraklinik.de (T.F.); ewilbers@barbaraklinik.de (E.W.); 2 Department of Neurosurgery, St. Barbara-Hospital, Academic Hospital of Westfaelische Wilhelms-University Muenster, 59073 Hamm, Germany; kyram@gmx.de (K.M.); cewelt@barbaraklinik.de (C.E.); 3 Department of Neurosurgery, Academic Hospital Koeln-Merheim, Witten/Herdecke University, 51109 Koeln, Germany; nakamuram@kliniken-koeln.de

**Keywords:** chronic subdural haematomas, coagulation disorders, haematoma width, recurrence, septation, surgical procedure

## Abstract

(1) **Background**: In this study, epidemiological, clinical, therapeutical, and haemostaseological variables were investigated regarding their correlation with the recurrence of chronic subdural haematomas to assess the risk of recurrence more reliably in everyday clinical practice. (2) **Methods**: In our retrospective study, the electronic records of 90 patients who underwent surgery for a chronic subdural haematoma at our institute between 1 January 2017 and 31 May 2021 were analysed regarding previously defined variables. (3) **Results**: In the patient collective, 33.33% of the 90 patients experienced a recurrence requiring treatment. The occurrence of a recurrence was not statistically significantly related to age, gender, known alcohol abuse, a specific location, extension over one or both hemispheres, the surgical method, or anticoagulant medication. However, the recurrence was statistically significantly related to haematoma width (*p* = 0.000007), septation (*p* = 0.005), and the existence of a coagulation disorder not treated with medication (*p* = 0.04). (4) **Conclusions**: In our study, the width of the haematoma, septation, and coagulation disorders not treated with medication were documented as risk factors for the occurrence of a chronic subdural haematoma. Identifying of these risk factors could help in adapting individual therapeutic concepts for chronic subdural haematomas.

## 1. Introduction

A subdural haematoma is a venous haemorrhage into the subdural space. As a result of the haemorrhage, a pathological subdural space forms, which causes a clinical picture due to compression [1]. A subdural haematoma is classified as chronic from the beginning of the third week after the causal event [2]. Various mechanisms contribute to the persistence and growth of chronic subdural haematomas. These include membrane formation, including the formation of new vessels in the outer membrane, fibrinolysis, and inflammatory processes. The outer membrane is not only important for vascularisation and permeability but also releases inflammatory mediators [3,4,5]. The use of anticoagulants and thrombocyte aggregation inhibitors, as well as congenital blood coagulation disorders, can be a favourable factor for the development of chronic subdural haematomas [6,7,8].

The symptoms of chronic subdural haematomas vary greatly. There can be asymptomatic courses but also seizures, memory disorders, headaches, and problems with movement and articulation [9,10]. Chronic subdural haematomas can have a long latency period before symptoms appear [11]. As part of the diagnosis, clinical symptoms must be combined with a detailed medical history, in which previous trauma in particular should be enquired about [10,11,12]. If the diagnosis is suspected, it must be confirmed by computerised tomography or magnetic resonance imaging of the skull [9]. The haematomas usually appear as hypodense concave–convex lesions on computed tomography [10,13].

Chronic subdural haematomas are usually treated surgically [14]. Current conservative treatment options include corticosteroids, tranexamic acid, statins, and osmotherapeutics such as mannitol [15,16,17,18]. Surgical treatment of chronic subdural haematomas involves one or two drill holes, a twist-drill craniotomy, or an osteoplastic craniotomy. In most cases, the subdural space is also irrigated with the insertion of a subdural drain. The aim of the procedure is to relieve the haematoma and thus eliminate the compression effect on the brain [19]. In the case of a recurrence requiring surgery, a surgical method is usually chosen that offers at least as much access and intraoperative overview as the primary operation [11]. Postoperative complications include recurrences, seizures, intracranial haemorrhages and haematomas, pneumocephalus, and failure of the brain to expand to the physiological size [10,20,21].

According to the literature, the recurrence rate for unilateral chronic subdural haematomas is 2.3% to 33% and 25% if a bihemispheric haematoma is present [11]. If there is a persistent accumulation of air postoperatively, this ensures the continuation of the pathological subdural space, in which blood can accumulate again and thus lead to a recurrence [22]. If there is a more complex haematoma structure with septa, complete irrigation, mobilisation, and removal of the haematoma are more difficult, which means that residual haematoma and fluid may remain. Incomplete haematoma removal and a firm inner membrane can promote re-bleeding into the subdural space and thus recurrence. The wider the chronic subdural haematoma, the greater the displacement and the necessary re-expansion of the brain parenchyma [23]. If age-related brain atrophy is present, this enables greater haematoma expansion and also leads to slower re-expansion of the brain after surgical haematoma relief [2,24].

The aim of our study is to investigate various epidemiological, clinical, therapeutical, and haemostaseological variables as well as radiological findings regarding their correlation with the occurrence of chronic subdural haematomas in order to be able to assess the risk of recurrence more reliably in everyday clinical practice and, if necessary, to adapt individual concepts of treatment accordingly.

## 2. Materials and Methods

The study protocol was conducted in accordance with the Declaration of Helsinki and approved by the ethics commission of the Medical Faculty, Witten/Herdecke University (Ref-Nr. 95/2020).

### 2.1. Patient Enrolment

This study is a retrospective study. Patients who were treated surgically for a subdural haematoma at our institute between January 2017 and May 2021 were included. Our clinic is a certified regional trauma centre in a region with a high density of neurosurgical clinics and thus with adequately covered neurotrauma services. Within our centre, there is a specialisation as a head centre with a sufficient level of specialist physicians, trained physician assistants, and dedicated nurse specialists. The prerequisites required in many studies exist in terms of a specialised neurosurgical intensive care unit, interventional neuroradiology, and structured shock room management with permanently available neurosurgical expertise [25]. In addition, the infrastructural conditions are in place to enable neurotraumatised patients to be quickly transferred to diagnostics and surgical treatment via the helipad and the shock room. 

In the study period, a total of 197 patients underwent surgical treatment for a chronic subdural haematoma, of whom 181 patients were referred from other clinics and 16 patients presented to our emergency department or outpatient clinic. Of this patient collective, 90 fulfilled the inclusion criteria. After surgical treatment, patients were referred to our neurosurgical practice for personal radiological and clinical follow-up care. Patients were selected based on the clinic’s internal data bank, in which ICD code I62.02 was defined as the inclusion criterion. Subdural hygromas, subdural haematomas with meningeosis carcinomatosa, or other pre-existing conditions were excluded in order not to distort the results due to influencing factors that were not investigated in detail.

The group of patients with a recurrence of a chronic subdural haematoma did not include those who showed a residual haematoma in the CT scan immediately after surgery, as this is not a classic recurrence by definition. The inclusion criterion was the occurrence of a recurrence requiring further surgical treatment within six months of the first operation.

### 2.2. Data Acquisition

The demographic variables include patient’s age and gender at the time of admission to hospital. The clinical variables listed in the dataset include alcohol abuse and the characteristics of the haematoma. In terms of localisation, a distinction was made between frontal, fronto-parietal, parietal, fronto-temporal, parieto-occipital location, and extension over the entire hemisphere based on the cortical landmarks of the brain surface [26].

The images were viewed and analysed using the JiveX image viewing software. The width of the subdural haematoma was measured in the axial plane at the punctum maximum using the measurement function integrated in the JiveX image viewing software. In the case of bihemispheric haematomas, the wider side was documented in each case. It was also indicated whether the haematoma extended over one or both hemispheres and whether septation was detected after imaging. The radiological images used to determine the indication for surgery were used for analysis.

As part of haemostaseology, the intake of anticoagulants and thrombocyte aggregation inhibitors was queried, and the name of the preparation used was included in the data record. Other diagnosed coagulation disorders were also included in the dataset. These were asked about and documented as part of the patient history. Furthermore, coagulation values from the laboratory tests performed were used for the data analysis. The first laboratory values recorded after hospitalisation were selected so that there was no falsification due to the administration of medication during the inpatient stay. Fibrinogen concentration, thrombocyte count, prothrombin time, and activated partial thromboplastin time were included in the dataset (Table 1).

### 2.3. Documentation of Surgical Procedures

The type of surgery conducted was tracked based on the surgical report and entered into the dataset. The surgical methods performed include borehole trephination, extended borehole trephination, osteoplastic craniotomy, and twist-drill craniotomy (Table 1). In the case of borehole trephination and twist-drill craniotomy, it was also documented whether one or two boreholes were involved. If bihemispheric subdural haematomas were identified and a twist-drill craniotomy or burr hole trephination was performed on both sides, only the larger subdural haematoma was considered and documented in the database in terms of width, and therefore only one burr hole was specified. In addition, it was documented whether irrigation of the subdural space and/or membrane opening was performed during the surgical procedure and whether a drainage was placed. In the patient group in which a recurrence occurred, it was also documented how often a recurrence occurred.

### 2.4. Statistical Analysis

The individual variables were documented in a dataset in the Microsoft Excel programme and then transferred to the statistical software IBM SPSS Statistics 27.0. A univariate analysis of the association of the various variables with the occurrence of a recurrence was carried out. For categorical variables, the chi-square test and Fisher’s exact test were performed. If more than two categorical variables were to be tested for a significant association, the Fisher–Freeman–Halton test was used. For non-categorical variables, the *t*-test was used to compare the mean values of the group of patients without a recurrence and those of the group of patients with a recurrence using hypothesis tests. In the multivariate logistic regression analysis, we initially conducted a regression analysis with the reoccurrence of subdural hematoma as the dependent variable against all other variables in an ANOVA. The obtained significance level was *p* < 0.001, confirming the validity of the analysis. Subsequently, a logistic regression model was employed to perform a multivariate analysis involving all variables. The relationship between the variables and the recurrence of chronic subdural hematoma is presented based on the 95% confidence interval.

## 3. Results

**Figure 1:**
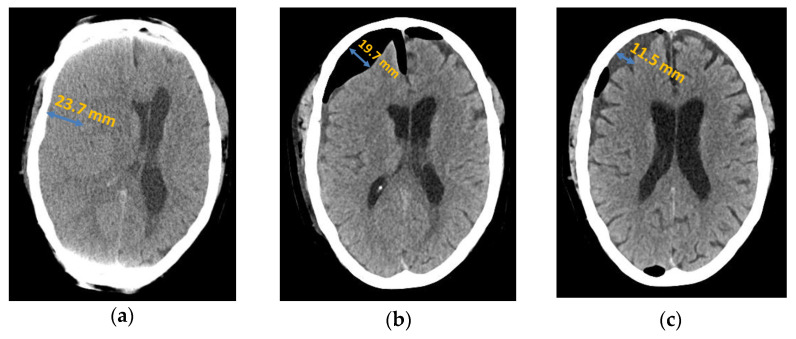
Axial computed tomography of chronic subdural haematoma: (**a**) preoperative presentation; (**b**) postoperative subdural air accumulation; (**c**) recurrence of chronic subdural haematoma.

The total patient population consisted of 90 patients with chronic subdural haematoma. In 29 patients (32%), no associated trauma could be identified. In 61 patients (68%), trauma in the weeks prior to diagnosis was identified as the cause of the subdural haematoma, including falls in 34 patients (38%), traffic accidents in 5 patients (6%), and minor trauma (e.g., minor head trauma to the edge of the door without a fall) in 22 patients (24%). Of the total patient population, 60 patients (67%) did not develop a recurrence and 30 patients (33%) had a recurrence. In 80% (*n* = 24) of these 30 cases, one recurrent haemorrhage required treatment, in 16.67% (*n* = 5) there was a second recurrence, and in 3.33% (*n* = 1), there was a third recurrence requiring treatment; see Figure 1.

### 3.1. Demographic Variables

The mean age of the patient group in which a recurrence occurred was 74.40 (±2.66) years, and this was 74.15 (±1.41) years in the patient group without recurrence. The comparison of the mean values using the *t*-test showed that there was no statistically significant difference (*p*-value = 0.93). Furthermore, the occurrence of recurrence showed no statistically significant correlation with the gender of the patients (*p*-value for “Fisher’s exact test” = 0.48).

### 3.2. Clinical Variables

#### 3.2.1. Alcohol Abuse

Alcohol abuse was only present in one patient (3.33%) in the group with recurrence and one patient (1.67%) in the group without recurrence. Although the percentage was higher in the recurrence group, no statistically significant correlation was found between the recurrence and alcohol abuse (*p*-value for “Fisher’s exact test” = 1.00).

#### 3.2.2. Characteristics of Haematoma

##### Width

In the patient group with a recurrence, the mean haematoma width was 22.83 mm (±1.31), whereas in the group without recurrence, it was 16.23 mm (±0.73). The *t*-test yielded a *p*-value of 0.000007, which showed that increasing haematoma size was statistically significantly related to the increased probability of recurrence of a chronic subdural haematoma. Table 2 shows an example of the statistical analysis of the data, in this case in relation to haematoma width.

##### Localisation

**Figure 2:**
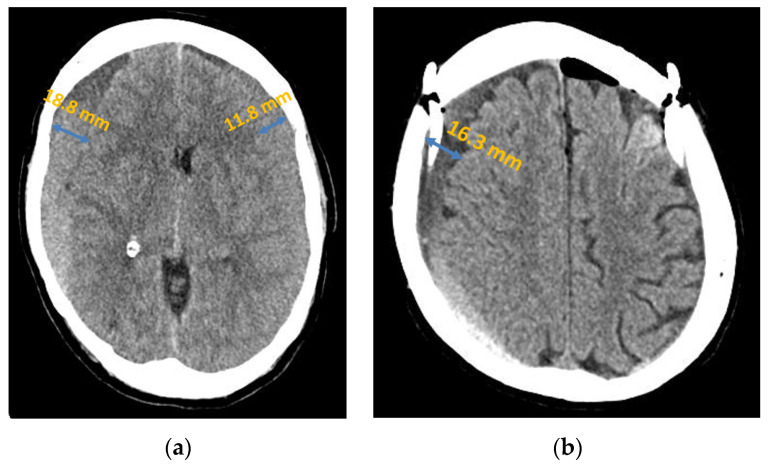
Chronic subdural haematoma extended over both hemispheres: (**a**) preoperative presentation in axial CT; (**b**) postoperative presentation in axial CT.

Considering the entire patient collective, the chronic subdural haematoma extended in most cases (45.56%, *n* = 41) over the entire affected hemisphere or over both affected hemispheres; see Figure 2. The second most common localisation was fronto-parietal localisation in 28.89% (*n* = 26), followed by frontal localisation in 16.67% (*n* = 15). This distribution followed the same order in both patient groups.

**Figure 3:**
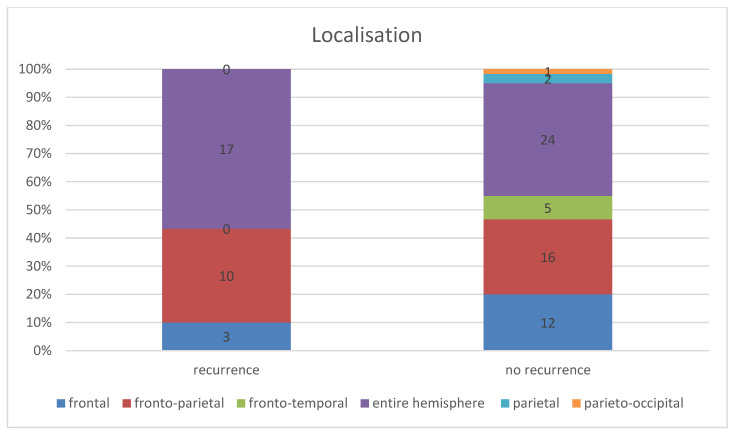
Comparison of haematoma localisation in the groups with and without recurrence.

In the patient group in which a recurrence occurred, the subdural haematoma extended over the entire affected hemisphere in 56.57% (*n* = 17), the fronto-parietal localisation in 33.33%, (*n* = 10), and the frontal localisation in 10.00% (*n* = 3). In the group of patients without a recurrence, 40.00% (*n* = 24) showed an extension over the entire hemisphere (16.56% lower than in the recurrent group of patients), 26.67% (*n* = 16) the fronto-parietal localisation, and 20.00% (*n* = 12) the frontal localisation. Furthermore, in two cases (3.33%), there was a parietal localisation and in one case (1.67%) a parieto-occipital localisation (Figure 3). However, the Fisher–Freeman–Halton exact test showed localisation was not a statistically significant prognostic factor for the risk of recurrence (*p*-value = 0.28). 

##### Septation

**Figure 4:**
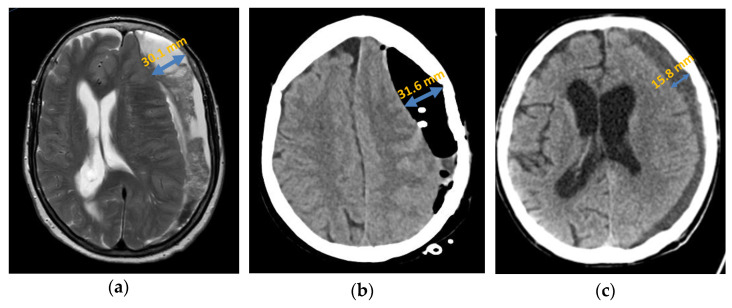
Axial presentation chronic subdural haematoma: (**a**) preoperative septation in axial plane in MRI; (**b**) postoperative subdural air accumulation in CT; (**c**) recurrence of chronic subdural haematoma in CT.

Primary septation of the chronic subdural haematoma was detected in 33.33% (*n* = 10) of patients with a recurrence either intraoperatively or by an imaging procedure (Figure 4). In the patient group with no recurrence, septation was detected in only 8.33% (*n* = 5). After analysis using Fisher’s exact test, which yielded a *p*-value of 0.005, septation was statistically significantly associated with an increased probability of recurrence in a chronic subdural haematoma. 

Generally, in the patients in whom septation was detectable, the recurrence rate was 66.67% (*n* = 10). However, in patients without detectable septation, the risk of recurrence was significantly lower at 26.67% (*n* = 20).

### 3.3. Surgical Procedure

#### 3.3.1. Surgical Method

Of the 90 haematomas documented in total, 74.44% (*n* = 67) were treated surgically with a burr hole trephination. The second most common procedure was an extended burr hole trephination (15.56%, *n* = 14). A craniotomy or twist-drill craniotomy was performed in 3.33% (*n* = 3) of haematomas. In two patients, two unilateral drill holes were chosen as the surgical treatment, which corresponded to 2.22% of the entire patient group. Furthermore, two unilateral twist-drill craniotomies were performed in one patient (1.11%). However, there was no statistically significant difference between the recurrence rates of the different surgical methods (*p*-value for “Fisher–Freeman–Halton test” = 0.55).

#### 3.3.2. Membrane Opening

In the patient group with a recurrence, the parietal membrane was opened intraoperatively in 16.67% (*n* = 5) of patients. In the patient group without a recurrence, this was only opened in 8.33% (*n* = 5) of cases (*p*-value = 0.29, non-significant). In the patient in whom the membrane was opened intraoperatively, the recurrence rate was 50.00% (*n* = 5), while it was 31.25% (*n* = 25) in the patients without membrane opening. However, this was not a statistically significant difference.

#### 3.3.3. Intraoperative Irrigation

Irrigation was performed in the first operation in 73.33% (*n* = 44) of patients who did not develop a recurrence and slightly more frequently at 76.67% (*n* = 23) in the group with a recurrence. However, the difference was not statistically significant (*p*-value for “Fisher exact test” = 0.80). In patients who underwent irrigation, a recurrence rate of 34.32% (*n* = 23) was observed. In those without irrigation, the recurrence rate was only slightly lower at 30.43% (*n* = 7).

#### 3.3.4. Subdural Drainage

A subdural drainage was inserted during the first surgical procedure in all patients in whom a recurrence was detected in the further course. In the patient group without a recurrence, only 1 out of 60 (1.67%) patients did not have a drainage inserted (*p*-value for “Fisher exact test” = 1.00, non-significant).

### 3.4. Haemostaseology

#### 3.4.1. Anticoagulant Medication

**Figure 5:**
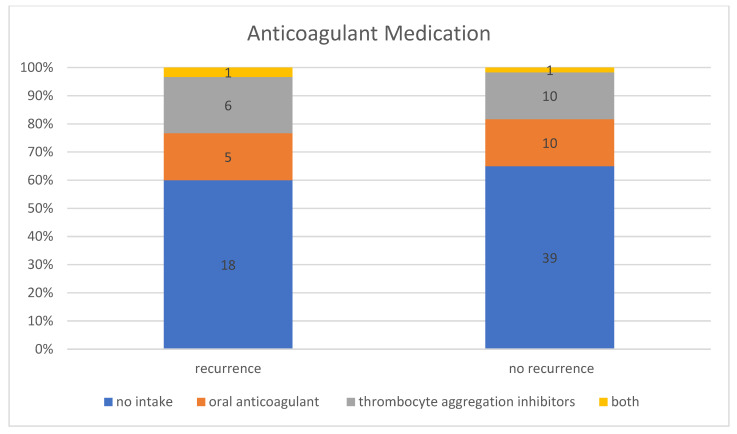
Intake of anticoagulant medication in patients with and without recurrence.

In the patient group without a recurrence, 35.00% (*n* = 21) of the 60 patients were taking anticoagulant medication, including 47.62% (*n* = 10) taking thrombocyte aggregation inhibitors, 47.62% (*n* = 10) taking oral anticoagulants, and 4.76% (*n* = 1) taking thrombocyte aggregation inhibitors and oral anticoagulants at the same time. In contrast, 40.00% (*n* = 12) of the patient group with a recurrence requiring treatment were taking anticoagulant medication, including 50.00% (*n* = 6) taking thrombocyte aggregation inhibitors, 41.67% (*n* = 5) taking oral anticoagulants, and 8.33% (*n* = 1) taking thrombocyte aggregation inhibitors and oral anticoagulant at the same time (Figure 5). However, this difference between the group with and without recurrence was not statistically significant (*p*-value for “Fisher–Freeman–Halton test” = 0.93). Therefore, no correlation could be established between the use of anticoagulant medication and an increased recurrence rate. In general, the recurrence rate was 36.36% (*n* = 12) in patients taking anticoagulant medication and 31.58% (*n* = 18) in patients with no known intake.

#### 3.4.2. Coagulation Disorders

In the group of patients who had a recurrence, three patients (10.00%) had known coagulation disorders. None of these patients had previously been treated with medication. Two of these patients (6.67%) suffered from thrombocytopenia, and one had an acquired coagulation factor deficiency. Among the patients who did not develop a recurrence, only one person had a coagulation disorder. This patient suffered from a hereditary factor V deficiency. Patients with a coagulation disorder not treated with medication had a statistically significantly increased recurrence rate, as the Fisher–Freeman–Halton exact test yielded a *p*-value of 0.04.

#### 3.4.3. Laboratory Parameters

The analysis of the coagulation values of laboratory tests performed showed no statistically significant difference between the two groups for any of the laboratory parameters analysed. 

The reference range for the fibrinogen concentration is 200–393 mg/dL. In the patient group with recurrence, the mean value of measured fibrinogen concentration at the time of admission was 344 ± 19 mg/dL. In the patients without a recurrence, this was slightly higher at 381 ± 17 mg/dL. However, this difference was not statistically significant (*p*-value for “*t*-test” = 0.19).

The mean value of the measured thrombocytes concentration in the group without a recurrence was 236 ± 9 thrombocytes/nL with a reference range of 150–450 thrombocytes per nanolitre; in the patient group with a recurrence, the mean value was lower at 213 ± 13 thrombocytes/nL. This difference was not statistically significant (*p*-value for “*t*-test” = 0.14). 

In the patient group without a recurrence, the mean prothrombin time measured on admission was 90% ± 2 with a reference range of 70–120%, whereas in the group with a recurrence, it was 85% ± 3 (*p*-value for “*t*-test” = 0.19, non-significant). 

In the patients without a recurrence, the mean value for the activated partial thromboplastin time was 29.9 ± 0.6 s with a reference range of 25 to 36 s, being slightly higher than the mean value in the group with a recurrence, which was 29.7 ± 0.9 s (*p*-value for “*t*-test” = 0.851, non-significant). Table 3 shows the results for the various variables analysed.

Altogether, the univariate analysis showed that haematoma width (*p* = 0.000007), septation (*p* = 0.005), and coagulation disorders (*p* = 0.04) were risk factors for recurrence of chronic subdural haematomas. Furthermore, the multivariate logistic regression analysis showed that haematoma width (*p* = 0.0001) and septation (*p* = 0.004) were independent risk factors for the recurrence of chronic subdural haematomas.

## 4. Discussion

In the present study, a recurrence rate of chronic subdural haematomas of 33.33% was found. Recurrence rates of 6.6% to 33% are reported in the literature [11,23,27]. 

### 4.1. Demographic Variables

The incidence of chronic subdural haematomas increases significantly with increasing age [6]. In the present study, the mean age of patient groups with and without recurrence differed only minimally. The average age was 74.15 years in the patients without a recurrence and 74.40 years in the group with a recurrence. These results are consistent with the increasing incidence found with increasing age described in the literature. This is attributed to more frequent fall events, the reduction in tissue stability, and increasing brain atrophy [2,24]. Another possible explanation for the increased incidence in old age is the reduced number of endothelial stem cells [28,29].

In the present study, no correlation between the gender of the patients and the probability of recurrence could be established, as in other studies [30]. However, there are studies in which a statistically significant higher recurrence rate was found in men [31]. One reason for the higher incidence in men is attributed to their more frequent multimorbidity [6]. Another reason could be the higher oestrogen levels in women [32]. However, it must be noted that chronic subdural haematomas occurs more frequently in older age and accordingly in the postmenopause.

### 4.2. Clinical Variables 

The proportion of patients with known alcohol abuse in our study was 2.22%. The patient collective in other studies indicates significantly higher proportions of 11.12%, for example [33]. Nevertheless, it can be assumed that patients with alcoholism have a higher probability of developing a chronic subdural haematoma due to more frequent falls, liver damage, and volume reduction of the brain [34].

Our study showed a statistically significant difference in the mean value of the haematoma width measured in the axial plane at the punctum maximum. These results are consistent with some of the studies in the literature [35]. This is primarily attributed to the greater displacement of brain parenchyma and the correspondingly greater necessary re-expansion of brain after haematoma relief [23]. On the other hand, in a study by Huang, Lin et al., there was no significant correlation between haematoma size and recurrence rate in the patient collective [36]. Due to these inconsistent findings, further studies with standardised volume calculation or size determination are required in order to clearly define the correlation.

In the present study, haematoma localisation was not a statistically significant prognostic factor for the risk of recurrence. Thus, the result is in line with the results of other studies [11,36]. However, the literature describes the extension of subdural haematomas over both hemispheres as a significant risk factor for recurrences in other patient collectives [37]. This could be due to the greater mean haematoma thickness in bihemispheric compared with unilateral chronic subdural haematomas found in a study by I.A. Iliescu [11].

In their study, Nanko, Tanikawa et al. found that subdural haematomas that are permeated by membranes have an increased concentration of vascular endothelial growth factors. These increase the formation of new vessels and consequently permeability and haematoma growth [4,38]. In our analysed patient groups, the recurrence rate in the patient group without detectable septation was statistically significantly lower than that in the patient group with septation. This was also confirmed by other studies [30,36]. However, opposite results were also found in individual studies [31]. The reason for the higher recurrence rate lies primarily in the more complex haematoma structure indicated by the septation. Accordingly, if the septation is already visible in the CT scan, a surgical method should be selected that allows extensive irrigation, mobilisation, and removal of the chronic subdural haematoma with a good overview.

Altogether, the results of our study, like the results of the “Oslo Chronic Subdural Hematoma Grading System for Prediction of Postoperative Recurrence Requiring Reoperation” study, showed in the analysis of clinical variables that the width of the haematoma, i.e., the haematoma volume, and the septation of the haematoma are the most important predictors for the occurrence of a recurrence requiring reoperation [39].

### 4.3. Surgical Procedure

In the patient population analysed, there was no statistically significant difference between the recurrence rates of chronic subdural haematomas and the different surgical methods used. This result is in accordance with a large majority of the results of other recent studies, in which no statistically significant difference in the recurrence rates of the various surgical methods was found [8,19,40,41]. However, individual studies contradict these results. Taussky, Fandino et al. compared the recurrence rates of patients who were treated with one drill hole with those of patients who were treated with two drill holes. A statistically significant higher recurrence rate was found in the group of patients who had only one drill hole trepanation (29%) compared with (5%) that in the group with two drill holes [42]. In contrast, Kansal, Nadkarni et al. found that the recurrence rates did not differ significantly between the patient groups with one or two drill holes [40]. Furthermore, Weigel, Schmiedek et al. were able to show that drill hole trephination offers a low risk of recurrence compared with twist-drill craniotomy, with a low morbidity rate compared with craniotomy [43]. However, if haematoma is already septated in imaging diagnosis, a craniotomy is recommended for a good intraoperative overview. The surgical method should be selected by the treating neurosurgeon depending on the characteristics of the haematoma [11]. It should be noted that the surgical method in the present study was selected by the treating neurosurgeon according to the respective haematoma structure.

In our study, the recurrence rate of patients who underwent membrane opening was higher than the recurrence rate of patients who did not undergo membrane opening. Intraoperative membrane opening may indicate a thick outer membrane, which in turn may indicate a septated and organised haematoma, as well as a thick inner membrane [23].

With regard to the correlation between the recurrence rate and irrigation within the surgical treatment of a chronic subdural haematoma, the results diverge [44]. No statistically significant difference in the recurrence rate between the groups was found within the analysed patient population in our study. Zakaraia, Adnan et al. came to the same conclusion in their study [45]. In contrast, Ishibashi, Yokokura et al. and Tahsim-Oglou, Beseoglu et al. found a reduced recurrence rate with additional irrigation [46,47].

As insertion of a subdural drainage is a routinely performed procedure in almost all patients with a chronic subdural haematoma at our institute, no well-founded comparison could be made in the present study between patients with and without drainage therapy with regard to the recurrence rate. Most of the studies mentioned in the literature found a significantly lower recurrence rate in patients undergoing drainage therapy [26,48]. In a study by Santarius, Kirkpatrick et al., a recurrence rate of 9.3% was found in the group with postoperative drainage and 24% in the group without drainage [27]. In contrast, a study by Ishibashi, Yokokura et al. found no significant difference in the risk of recurrence [46]. The duration of drainage therapy could also play a role. In a study by Yu, Han et al., a recurrence rate of 16.3% was found in patients with a drainage period of less than three days, whereas this was only 1.3% in the patient group with a prolonged drainage period of at least three days. A significant causal relationship was found. A drainage period of at least three days appears to be necessary in order to reduce the haematoma in the long term by normalising coagulation and fibrinolysis and to reduce the risk of recurrence [23].

### 4.4. Haemostaseology

The recurrence rate for patients who were taking anticoagulant medication was 36.36% compared with only 31.58% for patients with no known intake. This was not a statistically significant difference. The classification of the preparations into oral anticoagulant and thrombocyte aggregation inhibitors also revealed no statistically significant difference with regard to the recurrence rate. In the majority of studies, the use of anticoagulant medication was not found to be an independent risk factor for the development of recurrences of chronic subdural haematomas [30,31,37]. In contrast to these results, Wada, Yamakami et al. found that the use of thrombocyte aggregation inhibitors is associated with a significantly higher recurrence rate, with the risk of recurrence decreasing as the time between the last dose and the operation increases [49]. In the present study, anticoagulant medication was discontinued before the first operation and continued after the operation, usually for four weeks, in consideration of the risk assessment, which means that the data only allow an evaluation of the risk of recurrence to a limited extent.

In the present study, the existence of a coagulation disorder that was not treated with medication was associated with a statistically significant increase in the recurrence rate. The higher the bleeding tendency and the slower the haemostasis, the stronger and faster the described recurrent mechanism of a subdural haematoma can occur. 

The laboratory values examined did not prove to be significant risk factors for recurrences of chronic subdural haematomas. Some of the studies assume local hyperfibrinolysis, which triggers persistent bleeding from the outer membrane, while others assume systemic hyperfibrinolysis [4,50]. In our study, however, there was no low mean value of fibrinogen concentration in relation to the reference range of 200–393 mg/dL that would be in favour of systemic hyperfibrinolysis. In their study, Shim, Park et al. found significantly higher levels of fibrinogen cleavage products in the haematoma fluid compared with the patients’ plasma, thus supporting the hypothesis of local hyperfibrinolysis [51].

During the development and growth of chronic subdural haematomas, a hyperinflammatory and a low anti-inflammatory response can be detected at the same time. In a study by Stanisic, Aasen et al., a locally poorly organised immune response was observed [5]. Accordingly, no thrombocytosis, which could indicate an inflammatory process, was detected in the present study. 

The minimum prothrombin time of both groups was clearly below the reference range, which spoke in favour of an existing slowed coagulation in the sense of a slowed extrinsic activation of individual patients. However, the mean values showed that these were only marginal phenomena. The minimum activated partial thromboplastin time of 22.5 s for the entire patient collective was only 2.5 s below the reference range. At 59.7 s, however, the maximum was 23.7 s above the reference range. This also speaks in favour of slower coagulation in individual patients, albeit in the sense of a required intrinsic activation.

The blood level of endothelial stem cells is closely causally linked to the development and recurrence of chronic subdural haematomas [28]. A study by Song, Wang et al. found that patients who developed chronic subdural haematoma had a significantly lower number of endothelial stem cells than a healthy control group. Furthermore, the number of endothelial stem cells in patients who developed a recurrence was even lower than that in patients who did not develop a recurrence, resulting in a new therapeutic option for preventing recurrences [28]. Another treatment option is offered by statins, which, according to the current study situation, lead to faster resorption of the haematoma volume and a lower risk of recurrence [18].

## 5. Conclusions

In our study, the recurrence of subdural haematoma was not statistically significantly related to age, gender, known alcohol abuse, a specific location, extension over one or both hemispheres, the surgical procedure, or anticoagulant medication. However, haematoma size, primary septation of the haematoma, and existence of a coagulation disorder not treated with medication were statistically significantly related to the increased probability of recurrence of a subdural haematoma.

## Figures and Tables

**Table 1:** Analysed patients- and disease-related variables.

Variable	Unit	Selection Option
gender		male/female
age	years	
alcohol abuse		yes/no
haematoma width	mm	
localisation		frontal/fronto-parietal/parietal/fronto-temporal/parieto-occipital/hemispheric
hemisphere		hemispheric/bihemispheric
septation		yes/no
operation method		borehole trephination/extended borehole trephination/craniotomy/twist-drill craniotomy
membrane opening		yes/no
irrigation		yes/no
drainage		yes/no
thrombocyte aggregation inhibitors		yes/no
anticoagulants		yes/no
other blood coagulation disorders		yes (specify which coagulation disorder is involved)/no
fibrinogen	mg/dL	
thrombocyte count	amount/nL	
prothrombin time	%	
activated partial thromboplastin time	seconds	

**Table 2:** Comparison of haematoma width in patient groups with and without recurrence.

Statistical Analysis	Recurrence (unit in mm)	No Recurrence (unit in mm)
mean value	22.83	16.23
median	22.00	14.50
standard deviation of the mean	1.31	0.73
maximum	40.00	30.00
minimum	14.00	8.00
range	26.00	22.00
standard deviation	7.19	5.62

**Table 3:** Analysed epidemiological, clinical, therapeutical, and haemostaseological variables in patient groups with and without recurrence. * *p*-values < 0.05 are statistically significant.

Characteristics	Group 1 Recurrence (*n* = 30)	Group 2 No Recurrence (*n* = 60)	*p*-Value *
age (years), mean ± SD (min–max)	74.40 ± 2.66 (44–93)	74.15 ± 1.41 (46–97)	0.93
gender:			
male	22	38	0.48
female	8	22	
alcohol abuse	1 (3.33%)	1 (1.67%)	1.00
width (mm) ± SD (min–max)	22.83 ± 1.31 (14–40)	16.23 ± 0.73 (8–30)	0.000007
localisation:			
entire hemisphere	17 (56.57%)	24 (40.00%)	0.28
fronto-parietal	10 (33.33%)	16 (26.67%)	
frontal	3 (10.00%)	12 (20.00%)	
septation	10 (33.33%)	5 (8.33%)	0.005
surgical procedure:			
membrane opening	5 (16.67%)	5 (8.33%)	0.29
intraoperative irrigation	23 (76.67%)	44 (73.33%)	0.80
subdural drainage	30 (100%)	59 (98.33%)	1.00
haemostaseology:			
anticoagulant medication	12 (40.00%)	21 (35.00%)	0.929
coagulation disorders	3 (10.00%)	1 (01.66%)	0.04
laboratory parameters:			
fibrinogen concentration (mg/dL) ± SD	344 ± 19	381 ± 17	0.19
thrombocyte concentration(thrombocyte/nL) ± SD	213 ± 13	236 ± 9	0.14
prothrombin time	85% ± 3	90% ± 2	0.19
activated partial thromboplastin time (seconds)	29.7 ± 0.9	29.9 ± 0.6	0.851

## Data Availability

Data are contained within the article.
